# Structural Basis and Kinetics of Force-Induced Conformational Changes of an αA Domain-Containing Integrin

**DOI:** 10.1371/journal.pone.0027946

**Published:** 2011-11-28

**Authors:** Xue Xiang, Cho-yin Lee, Tian Li, Wei Chen, Jizhong Lou, Cheng Zhu

**Affiliations:** 1 School of Life Sciences, Sun Yat-sen University, Guangzhou, China; 2 Wallace H. Coulter Department of Biomedical Engineering, Georgia Institute of Technology and Emory University, Atlanta, Georgia, United States of America; 3 Department of Oncology, National Taiwan University Hospital, Taipei, Taiwan; 4 Laboratory of Noncoding RNA, Institute of Biophysics, Chinese Academy of Sciences, Beijing, China; University of Bergen, Norway

## Abstract

**Background:**

Integrin α_L_β_2_ (lymphocyte function-associated antigen, LFA-1) bears force upon binding to its ligand intercellular adhesion molecule 1 (ICAM-1) when a leukocyte adheres to vascular endothelium or an antigen presenting cell (APC) during immune responses. The ligand binding propensity of LFA-1 is related to its conformations, which can be regulated by force. Three conformations of the LFA-1 αA domain, determined by the position of its α_7_-helix, have been suggested to correspond to three different affinity states for ligand binding.

**Methodology/Principal Findings:**

The kinetics of the force-driven transitions between these conformations has not been defined and dynamically coupled to the force-dependent dissociation from ligand. Here we show, by steered molecular dynamics (SMD) simulations, that the αA domain was successively transitioned through three distinct conformations upon pulling the C-terminus of its α_7_-helix. Based on these sequential transitions, we have constructed a mathematical model to describe the coupling between the αA domain conformational changes of LFA-1 and its dissociation from ICAM-1 under force. Using this model to analyze the published data on the force-induced dissociation of single LFA-1/ICAM-1 bonds, we estimated the force-dependent kinetic rates of interstate transition from the short-lived to intermediate-lived and from intermediate-lived to long-lived states. Interestingly, force increased these transition rates; hence activation of LFA-1 was accelerated by pulling it via an engaged ICAM-1.

**Conclusions/Significance:**

Our study defines the structural basis for mechanical regulation of the kinetics of LFA-1 αA domain conformational changes and relates these simulation results to experimental data of force-induced dissociation of single LFA-1/ICAM-1 bonds by a new mathematical model, thus provided detailed structural and kinetic characterizations for force-stabilization of LFA-1/ICAM-1 interaction.

## Introduction

Integrins are a family of heterodimeric transmembrane receptors composed of an α and a β subunit that involve in a wide variety of physiological processes such as cell adhesion, cell migration and immunoresponse [1]. They usually bear forces upon binding to ligands in cell-cell and cell-extracellular matrix adhesions, which are crucial to mechanosensing and mechnotransduction of cells [2], [3]. Of the 24 known human integrins, 10 of them, including the integrin α_L_β_2_ or lymphocyte function-associated antigen 1 (LFA-1) studied here, have an additional αA (or αI) domain inserted in the β-propeller domain of the α subunit, where the ligand binding site resides [4]. By binding intercellular adhesion molecule 1 (ICAM-1), LFA-1 mediates adhesion of leukocytes to the blood vessel wall or antigen presenting cells (APC), and sustains forces generated by the blood flow or the cell's motile machinery [1], [5].

**Figure 1:**
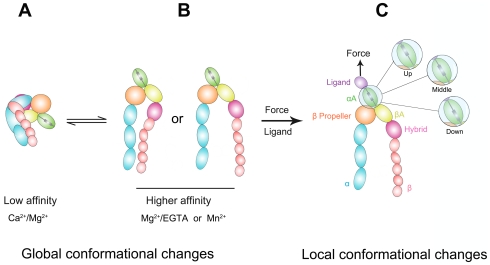
Conformational changes of αA domain-containing integrin. (A, B) Global conformations of integrin are affected by cation conditions. The integrin assumes a bent conformation with a low ligand binding on-rate under Ca^2+^/Mg^2+^ (A). In Mg^2+^/EGTA or Mn^2+^, the conformation may be shifted to an extended form with a closed (left) or swung-out (right) hybrid domain and correspondingly higher ligand binding on-rates (B). (C) Force applied via a bound ligand switches the αA domain from closed (α_7_-helix at the up position), intermediate (α_7_-helix at the middle position), and open (α_7_-helix at the down position) conformations with different off-rates.

In response to various biochemical [3], [4], [6] and mechanical signals [7], [8], integrins change conformations and ligand binding affinities. In physiological condition, they may assume a bent conformation and have a low ligand binding affinity. Inside-out signaling or changes in the metal ion conditions from Ca^2+^/Mg^2+^ to Mn^2+^ result in integrin conformational change to an extended form, with a closed or swung-out hybrid domain, accompanied by a higher ligand binding affinity (Fig. 1A, 1B) [3], [4], [9].

In addition to global conformational changes in the whole ectodomain and in the hybrid domain, the αA domain conformation also controls the affinity of αA-containing integins such as LFA-1 [5], [9]. Several αA domains, including that of LFA-1, have been crystallized [10]–[15], revealing as many as three conformations termed closed, intermediate and open, depending on the position of the C-terminal α_7_-helix [5] (Fig. 1C). As measured by surface plasmon resonance [5] and micropipette adhesion frequency assay [6], LFA-1 with the αA domain locked in the intermediate and open conformations have hundreds and thousands folds higher affinities for ICAM-1, respectively, than that locked in the closed conformation. A study of molecular dynamics (MD) simulations of αA domains with implicit water suggested that the fractions of these three conformation states are sensitive to the force applied to the C-terminus of their α_7_-helix [16]. Using a biomembrane force probe (BFP), single LFA-1/ICAM-1 bonds are found to dissociate from three states with distinct apparent off-rates and associated fractions [9]. The short-lived fraction (with the greatest apparent off-rate) is dominant at zero force and the fractions of intermediate- and long-lived states increase with the tensile force applied to the bond. The force-dependent transitions among these three fractions of bond states give rise to the LFA-1/ICAM-1 catch bond behavior in which the bond lifetimes are prolonged by tensile force in a certain regime [9].

Building from the above studies, we used steered molecular dynamics (SMD) simulations with explicit water to study the force-induced transitions of conformations of the LFA-1 αA domain. We also constructed a mathematical model to describe the interstate transitions integrin and their coupling with ligand dissociation. Using this model, we re-analyzed our previous data on single LFA-1/ICAM-1 bonds lifetimes measured from biomembrane force probe (BFP) force-clamp experiments [9], and estimated interstate transition rates that govern the time courses for activation of the liganded LFA-1 under force [9].

## Results

### SMD-simulated force-induced conformation transitions of LFA-1 αA domain

**Figure 2:**
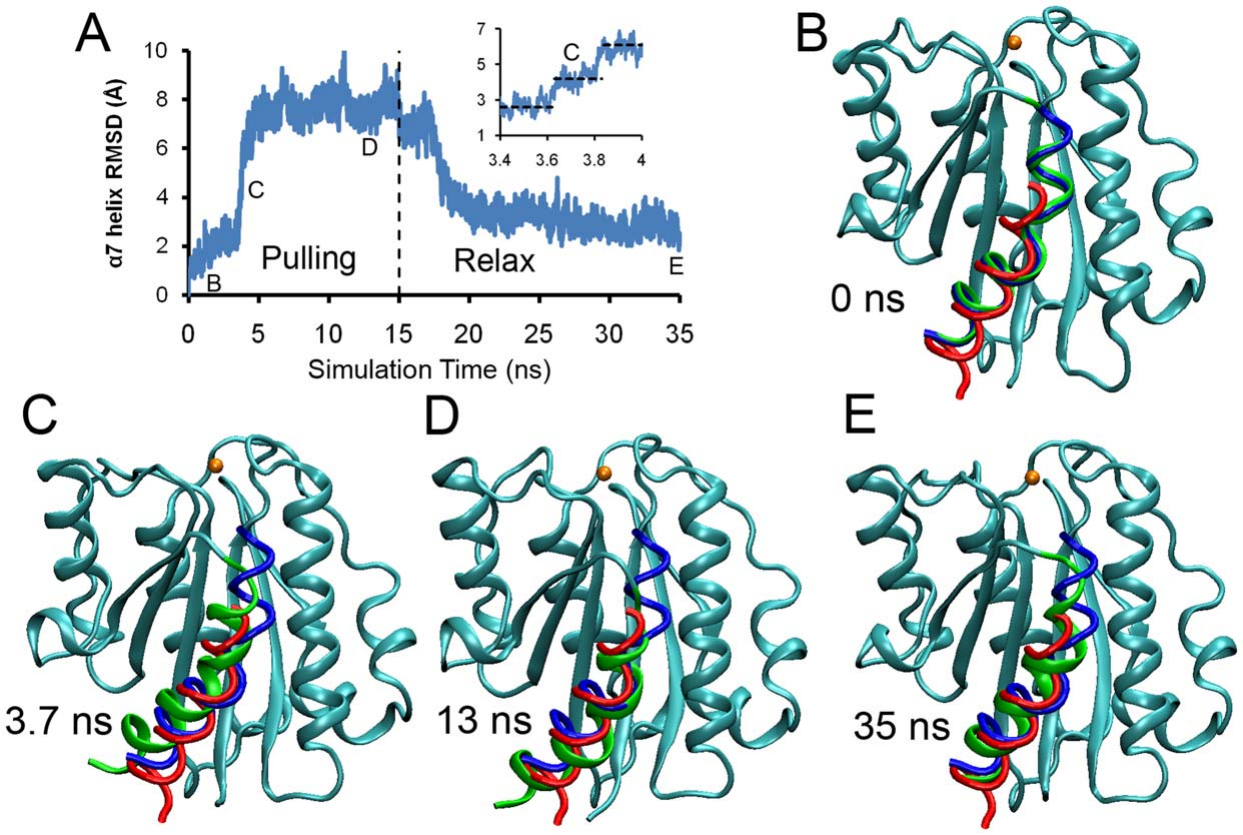
SMD simulation of pulling the α_7_-helix of the LFA-1 αA domain. (A) The time course of the RMSD between the simulated α_7_-helix structure and the equilibrated structure at the up position. Letters indicate time points where the snapshots shown in *B–E* were taken. Dashed vertical line marks the time when force was released to allow free dynamics simulation. The inset shows the successive conformational changes through three stable conformations marked with dashed lines at 3.4–4.0 ns. (B–E) Snapshots of the simulated structures (cyan) at the indicated times with the α_7_-helix shown in green. The equilibrated α_7_-helix at the up (blue) and down (red) positions are also shown. The Mg^2+^ ion is shown as golden spheres. See also Figure S1 and Video S1.

To study the force-induced conformational transitions of the LFA-1 αA domain, we used constant-force SMD simulations to pull the C-terminus of its α_7_-helix, as the position of the tension-bearing α_7_-helix determines the αA domain conformation [5], [16]. Unlike the previous implicit water simulations [16], our simulations included physiologically relevant water molecules. To observe the sequential transitions of the α_7_-helix position, we quantified the root mean square distance (RMSD) between the simulated structure and its initial “up” position, which corresponds to the “closed” conformation of the αA domain [16]. Pulling the α_7_-helix C-terminus in the first 3.6 ns only increased the RMSD slightly, indicating the stability of the “up” position (Fig. 2A, 2B). A sudden increase of the RMSD from 3 to 6 Å was then observed during 3.4–4 ns simulations, suggesting state transitions. Zooming in this transition phase with a magnified time scale, a stable “intermediate” α_7_-helix position with a 4.5-Å RMSD was observed (Fig. 2A inset, 2C). This “intermediate” α_7_-helix position is linked to the “intermediate” conformation of the αA domain. After two abrupt increments, the RMSD was stabilized at around 8 Å for the next 10 ns, corresponding to a “down” position of the α_7_-helix and the “open” conformation of the αA domain (Fig. 2A, 2D). After the pulling force was removed at the 15 ns time point, the α_7_-helix returned back from the “down” position to the “up” position in a few nanoseconds and remained up within the next 20-ns simulations (Video S1).

Besides the α_7_-helix position, another remarkable difference between the open and closed conformation of LFA-1 αA domain revealed by structural studies is the metal ion position at the metal ion dependent adhesion site (MIDAS). It was observed that in the open conformation, the MIDAS metal ion underwent inward movement for about 2 Å. Previous implicit water molecular dynamics simulations suggested that the movement of α_7_-helix and that of the MIDAS metal ion were coupled. Hence, we measured the RMSD of the MIDAS metal ion and other important residues between the simulated structures and the open or closed conformations (Figure S1). These included residues S139, S141, T206, and D239 that coordinated the MIDAS metal ion and residues L289, F292, and L295 that formed a “ratchet”-like structure to define the position of the α_7_-helix. In the simulations, although the pulling force induced movements of the α_7_-helix, no movements of the MIDAS metal ion were observed (Figure S1B), nor were their coordinating residues (Figure S1C–F). Nevertheless, we did observe the relevant “ratchet”-like movements on residues L289, F292, and L295 (Figure S1G–I), which followed the movements of the α_7_-helix.

**Figure 3:**
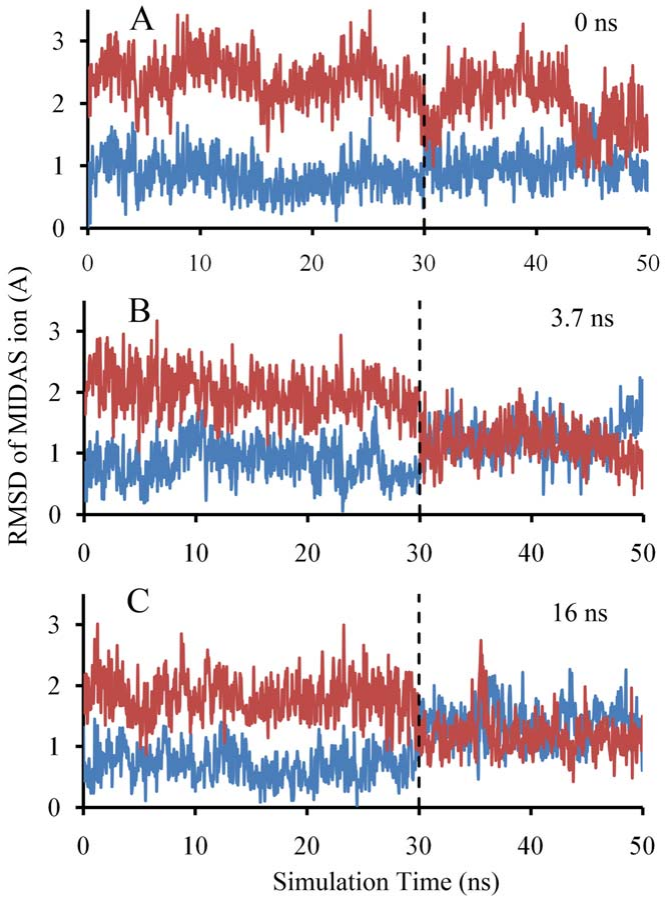
Simulated inward movements of the MIDAS metal ion. RMSD time courses of the MIDAS metal ion between the simulated structure and its closed or open positions were shown in blue or red, respectively. The simulations started from the snapshots at 0 ns (A), 3.7 ns (B) and 16 ns (C) of the SMD simulations described in Fig. 2. Dashed vertical line marks the time when the point charges of the two carboxyl oxygens of residue D239 were reduced by 0.5*e* each.

Residue D239 coordinated directly with the MIDAS metal ion in the closed conformation as observed in the crystal structures [11], [12]. On the other hand, in the open conformation, D239 might not coordinate with metal ion directly but through a water molecule. In our pulling simulation, it seemed that the strong ionic interaction between D239 and the metal ion constrained the metal ion at its closed (outward) position, thus preventing the inward movement from being observed within the short timescale of the simulation. To test this hypothesis, we performed a set of three simulations. These simulations started from the structures generated from the above pulling simulations. The snapshots at 0, 3.7 and 16 ns were taken as the respective new starting points. Among them, the 0 ns configuration represented the “up” position of the α_7_-helix, the 3.7 ns configuration represented the “middle” position and the 16 ns one represented the “down” position. In these free dynamics simulations, the applied force was released. To prevent the α_7_-helix from returning back to the “up” position in the simulations starting from 3.7 and 16 ns snapshots, we constrained the Cα atoms of the α_7_-helix in addition to the original constraint residues. Firstly, 30 ns free dynamics simulations were performed followed by 20 ns free dynamics simulations with the point charges of the two oxygen atoms of D239 carboxyl group reduced by 0.5*e* each. As shown in Fig. 3 with the RMSD time courses of the MIDAS ion between the simulated structure and its closed or open positions, in all three simulations, the MIDAS ions fluctuated around their closed position without any tendency to move towards the open position before the point charges were reduced. By comparison, after the point charges of the D239 carboxyl oxygen were reduced, in the simulations starting from 3.7 ns (α_7_-helix at middle position) and 16 ns (α_7_-helix at down position) (Fig. 3B and 3C), the metal ion showed strong tendencies to move inward towards the open position, with the RMSD to the closed position reduced and that to the open position increased. For the simulation starting from 0 ns (α_7_-helix at down position) (Fig. 3A), the movement was also possible (30–32 ns and 44–46 ns in Fig. 3A), but the duration was short. The simulated structure fluctuated around the closed position for the majority of simulation times. These simulations confirm that the position of the metal ion is related to the position of the α_7_-helix, consistent with the generally accepted contention that the position of the metal ion determines the ligand binding affinity of the αA domain.

These results support the hypothesis that the closed, intermediate and open conformations of LFA-1 αA domain represent stable states and that sequential transitions from the closed to intermediate and from intermediate to open conformations can be induced by pulling the α_7_-helix.

### Mathematical model for force-induced interstate transition of LFA-1 and ICAM-1 dissociation

**Figure 4:**
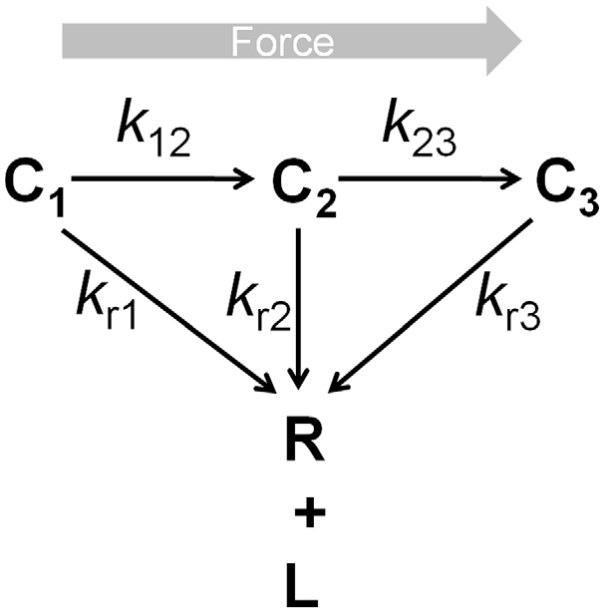
Schematic of the kinetic model of coupled conformational change of LFA-1 and dissociation from ICAM-1 under force. A LFA-1/ICAM-1 bond is assumed to dissociate irreversibly from one of three states – C_1_, C_2_ and C_3_ – with respective reverse-rates *k*_r1_, *k*_r2_ and *k*_r3_. This is coupled with one-way sequential transitions from C_1_ to C_2_ and from C_2_ to C_3_ with respective interstate transition rates *k*_12_ and *k*_23_. The corresponding mathematical model is Equations 1–3 in Materials and Methods.

Our SMD simulations suggested that the LFA-1 αA domain transitioned from the closed, intermediate and open conformations successively by applied force (Fig. 2). To incorporate such conformational change kinetics into the kinetics of force-induced ligand dissociation, we constructed a mathematical model for the BFP force-clamp experiment in which single LFA-1/ICAM-1 bonds were pulled with a constant force until rupture [9]. This simple model considers two interstate transition steps: from state C_1_ to state C_2_ and from state C_2_ to state C_3_ (Fig. 4) as well as three ligand dissociation steps from each of the three states. Each of these steps is assumed irreversible, which seems reasonable under force, as force drives unidirectionally both the interstate transition and ligand dissociation.

**Figure 5:**
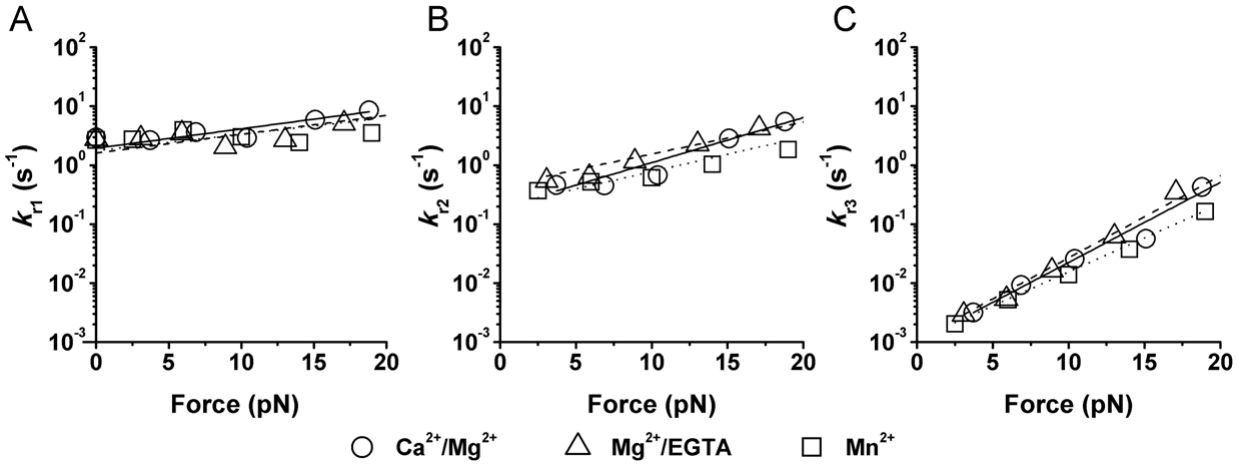
Force-dependent reverse-rates of three states under different cation conditions. Intrinsic reverse-rates *k*_r1_ (A), *k*_r2_ (B) and *k*_r3_ (C) of ICAM-1 dissociating from respective LFA-1 states C_1_, C_2_, and C_3_ (see Fig. 3) were estimated by fitting the experimental data from Ref. [9] with our kinetic model (equations 22–24) and plotted versus force at indicated cation conditions.

**Figure 6:**
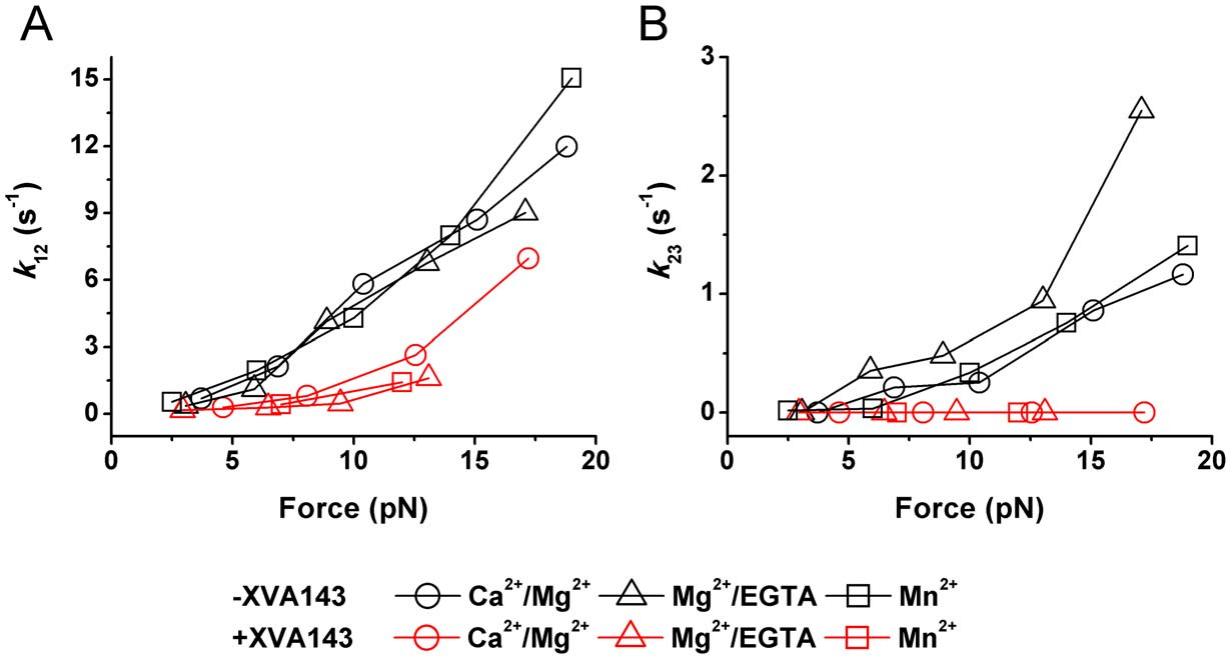
Force-dependent interstate transition rates. (A) The rate of transition of ICAM-1-bound LFA-1 from short- to intermediate-lived states (*k*_12_) is accelerated by force. This force-accelerated transition rate is suppressed by XVA143 (red). (B) The transition rate of ICAM-1-bound LFA-1 from intermediate- to long-lived states (*k*_23_) is accelerated by force, which is nearly completely blocked by XVA143 (red). The force-dependency of transition rates is not affected by cation conditions.

**Figure 7:**
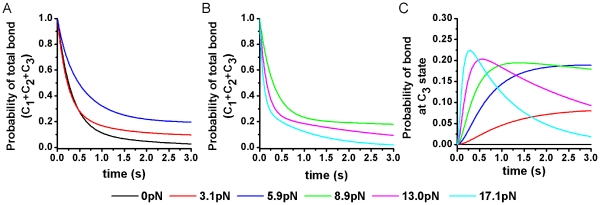
Predicted time courses of LFA-1/ICAM-1 bond survival probability at different forces. (A and B) The total survival probability of LFA-1/ICAM-1 bond (sum of all three states) decayed slower as the force increased from 0 to 5.9 pN (A) and decayed faster as force increased further (B). (C) The force-dependent time courses of the survival probability of the long-lived state (C_3_). The presence of C_3_ state was induced by force, indicating activation of LFA-1 by force applied via the ICAM-1 bond. As force increased from 0 to 17.1 pN, the time needed to reach the maximal probability was shortened with the maximum level increased, indicating shorter activation time with higher activity. Data obtained in Mg^2+^/EGTA were taken as representative parameters for the model prediction.

The model results in a set of coupled, linear, first-order, ordinary differential equations (Equations 1–3, Materials and Methods) governing the changes of the probabilities of the LFA-1/ICAM-1 bond in the three states in time, with constant coefficients (functions of force but not time): two interstate transition rates, *k*_12_ and *k*_23_, as well as three reverse-rates *k*_r1_, *k*_r2_ and *k*_r3_. The equations were solved analytically (Equations 7–9, Materials and Methods). The solution was fit to the data of the BFP force-clamped experiments [9] to obtain three apparent dissociation rate constants *k*_1_, *k*_2_, *k*_3_ and their associated apparent fractions ***ω***_1_, ***ω***_2_, ***ω***_3_ (summarized in Tables S1,S2,S3,S4,S5,S6). The intrinsic parameters are expressed as functions of the apparent parameters (Equations 20–24, Materials and Methods) and evaluated at different forces. Since the unique force-stabilizing catch-bond behavior of LFA-1/ICAM-1 interaction occurred at the force regime of about 10 pN, only the data below 20 pN were analyzed and shown (Figs. 5,6,7).

### Analysis of force-dependent ICAM-1 dissociation reveals characteristics of three LFA-1 states

The intrinsic reverse-rates, *k*_r1_–*k*_r3_, of ICAM-1 dissociation from the three LFA-1 states were plotted versus force in Fig. 5 in the range analyzed. They follow trends similar to the apparent off-rates determined previously [9], but are quantitatively different. Interestingly, ICAM-1 dissociated from state C_1_ with the highest but least force-sensitive reverse-rate *k*_r1_ (Fig. 5A), from state C_2_ with an intermediate reverse-rate *k*_r2_ that has an intermediate force sensitivity (Fig. 5B), and from state C_3_ with the lowest but most force-sensitive reverse-rate *k*_r3_ (Fig. 5C). Although the model assumes that all bonds start from state C_1_ and then proceed successively to states C_2_ and C_3_, the *k*_r1_–*k*_r3_ values were evaluated from data without assuming *a priori* their relative values and relative sensitivities to force. It is therefore gratifying that our analysis of the previous BFP experimental data [9] with the present model returns the results that state C_1_ is short-lived, state C_2_ is intermediate-lived, and state C_3_ is long-lived. These results indicate a correlation between the experimentally observed short-, intermediate- and long-lived states of LFA-1/ICAM-1 bonds and the SMD-simulated closed, intermediate, and open conformations of the LFA-1 αA domain (Fig. 2).

The force dependencies of all these intrinsic reverse-rates follow the Bell model [17], as indicated by the linear reverse-rates vs. force semi-log plots. They were indifferent to cation conditions Ca^2+^/Mg^2+^ Mg^2+^/EGTA or Mn^2+^, suggesting that the initial global conformation of the LFA-1 before it was liganded and stressed did not affect the intrinsic dissociation rates.

### Force-dependent kinetics of LFA-1 transitions from short- to intermediate- to long-lived states and inhibition by XVA143

Interestingly, our kinetic analysis found that the transition rate *k*_12_ from the short- to intermediate-lived states of LFA-1/ICAM-1 bonds (Fig. 6) was zero at zero force but increased with force in the range studied (Fig. 6A). Force also enhanced the transition rate *k*_23_ from the intermediate- to long-lived states of LFA-1/ICAM-1 bonds from its zero value at zero force (Fig. 6B), but to a lesser extent (compare the two Fig. 6 panels). In the force regime studied (<20 pN), the force-dependent interstate transition rates were indifferent to the cation conditions Ca^2+^/Mg^2+^, Mg^2+^/EGTA or Mn^2+^, thus were not affected by the initial global conformation of the LFA-1 molecule before it was liganded and stressed.

With XVA143, a small molecule antagonist that blocks the interaction between the αA and βA domains [18], [19], the force-dependent *k*_12_ was suppressed (Fig. 6A, red). The transition from the intermediate- to long-lived states of LFA-1 was nearly completely blocked by XVA143, as shown by the zero *k*_23_ in the force range studied (Fig.6B, red). A possible explanation for this result may be that the force applied on the α_7_-helix to induce the conformational changes has to be transmitted through the connection between αA and βA domains. Further, the suppression and blocking effects of XVA143 on *k*_12_ and *k*_23_ were not affected by the cation conditions.

### Force decelerates LFA-1 dissociation from ICAM-1 by accelerating LFA-1 activation

With the intrinsic parameters *k*_12_, *k*_23_, and *k*_r1_–*k*_r3_ estimated, we used equations 1–3 to study the dynamic evolution of LFA-1/ICAM-1 bonds and of individual conformation states and their overall behavior. As shown with representative model predictions for the Mg^2+^/EGTA condition, ligand dissociation manifests as decrease in time of the total survival probability of an LFA-1/ICAM-1 bond in all three states (Fig. 7 A and B). The decay of the curve is decelerated by force from 0 to 5.9 pN (Fig. 7A). This is not surprising since this force range corresponds to the experimentally observed catch-bond regime where the bond lifetimes are prolonged by force [9]. As force further increases, the decay of the bond survival probability is accelerated by force (Fig. 7B), corresponding to the slip-bond regime where the bond lifetimes are shortened by force, also observed experimentally [9]. Similar trends are predicted for other cation conditions (data not shown).

Remarkably, our analysis predicts that as force increases, the probability vs. time curves of the long-lived LFA-1/ICAM-1 bonds (C_3_ state) are left-shifted, as the slope of the initial phase is increased and the time needed to reach the maximal probability is shortened by 10-folds, from >3 s to ∼0.3 s (Fig. 7C). Since LFA-1 with an open αA domain binds ligand with the highest affinity [5] and the lowest reverse-rate (Fig. 5C), this result indicates that force accelerates the activation of LFA-1/ICAM-1 bond by increasing the interstate transition rates.

## Discussion

As primary force-bearing molecules governing cell-cell and cell-matrix adhesions [2], [3], integrins are tightly regulated biochemically [3], [4], [6] and mechanically [7], [8] via their dynamic conformational changes. The closed, intermediate and open conformations of the integrin LFA-1 αA domain metal ion dependent adhesion site (MIDAS) have been observed crystallographically to couple with the up, middle and down positions of its α_7_-helix position [5]. The distribution among these conformations has been observed by MD simulations to depend on force [16]. The present work has added to this body of literature by defining the sequential process of the force-induced conformational changes of the LFA-1 αA domain and modeling the coupled kinetics of interstate transition between, and ligand dissociation from, different LFA-1 states.

Unlike the previous implicit water SMD study that analyzed the cluster distribution of αA domain conformations at the end of force application [16], our explicit water SMD simulations have observed the sequential transitions of the αA domain under force: Upon pulling the LFA-1 αA domain C-terminus, the α_7_-helix successively moved from the up to middle and down positions (Fig. 2). Our reduced charge simulations suggest that when α_7_-helix stays in middle or down position, the MIDAS ion has a strong tendency to move inward to its open position, which binds ligand with high affinity. These simulations indicate that applied force results in successive changes from the closed to intermediate and open conformations.

The force-induced transition of the three αA domain conformations observed in our simulations correlates with the force-dependent three-state dissociation observed in our previous BFP experiment [9]. Another interesting simulation result is that the α_7_-helix relaxed back to the up position after force removal (Fig. 2A), suggesting that force is required to maintain its intermediate and down conformations under the simulation conditions. This also correlates with the experimental observation that the LFA-1/ICAM-1 reverse-rate at zero-force was indifferent to changes in cation conditions and XVA143 treatment [9]. These correlations support our hypothesis that while the ICAM-1 association on-rate depends on the global conformations of LFA-1, the ligand dissociation off-rate is primarily determined by the αA domain conformation, which has been supported by experiment [9].

We constructed a mathematical model to further test this hypothesis, by examining how the three αA domain conformational transition may be related to the three-state dissociation kinetics. The model assumes force-induced successive transitions from C_1_ to C_2_ and C_3_ states (Fig. 4), in accordance with the SMD results. Comparing to the previous phenomenological treatment, which fitted the force-dependent lifetime distributions by three apparent off-rates and their associated static fractions [9], the present mechanistic model treats the coupled kinetics of both interstate transition and ligand dissociation. This new model advances our knowledge in several aspects.

First, analyzing the previous BFP experiments [9] with this model has shown that the stability of LFA-1/ICAM-1 bonds are lowest at C_1_, intermediate at C_2_, and highest at C_3_ states, suggesting a correspondence of the short-, intermediate- and long-lived states with the closed, intermediate, and open conformations, respectively. Incorporating other forms of integrin conformational changes and relating them to functionality will be an important subject of future studies.

Second, the previously proposed allosteric mechanism for the LFA-1/ICAM-1 catch-slip bond [9] can be fully accounted for using the newly evaluated intrinsic parameters. Indeed, although the force-dependent dissociation of ICAM-1 from each of the three states behaves as slip bonds (Fig. 5), force accelerates transition from C_1_ to C_2_ more than it does dissociation from C_1_ to R+L (compare Figs. 5A and 6A). Force also increases transition rate *k*_23_ from C_2_ to C_3_ comparably to it does dissociation rate *k*_r2_ from C_2_ to R+L (compare Figs. 5B and 6B). This interplay between force-accelerated interstate transition and dissociation gives rise to the LFA-1/ICAM-1 catch bond at low forces (Fig. 7A) and slip bond at higher forces (Fig. 7B), as observed experimentally [9].

Third, our model reveals that XVA143 suppresses the transition from C_1_ to C_2_ and inhibits the transition from C_2_ to C_3_ without altering the intrinsic reverse-rates *k*_r1_–*k*_r2_ for dissociation from the three LFA-1/ICAM-1 bond states. This result has elucidated the mechanism for XVA143 to covert the LFA-1/ICAM-1 catch-slip bond to slip-only bond. Because both interstate transitions are induced by force (Fig. 6), our data indicate that XVA143 significantly weakens the force transmission from the αA to βA domains by blocking the binding of the intrinsic ligand of the αA domain α_7_-helix to the βA domain MIDAS [18], [19]. This finding supports the hypothesis that the three-state dissociations of LFA-1/ICAM-1 bonds are tightly regulated by the three-conformation transition of the LFA-1 αA domain.

Fourth, the new model has allowed us to estimate the time scale for integrin activation by force. Integrin activation has been suggested to be almost instantaneous [3], but data from different experiments are variable. Binding of fluorochrome-labled ligands to integrin α_IIb_β_3_ reveals fast reversible formation of an integrin/ligand precomplex followed by a stable irreversible complex, during which the affinity upregulation occurs in a time scale of 10 seconds [20], [21]. Conversion from selectin-mediated rolling to integrin-mediated firm adhesion of leukocytes on endothelium and the detachment followed thereafter are used as criteria for integrin activation and deactivation [3], [22], [23]. Chemokine-triggered full activation of LFA-1 mediates arrest of rolling lymphocytes on high endothelial venules within 1 second under flow conditions similar to those in the circulation [3], [24]. The conversion of rolling to stationary adhesion after the initial attachment of a neutrophil is induced by IL-1 in as little as 0.24 s in the presence of 1 dyn/cm^2^ shear stress [22]. Force has been shown to facilitate the affinity upregulation at the cellular level. Our work provided the first estimates at the single-molecule level for the time scales of force-induced integrin activation from the reciprocal interstate transition rates, 1/*k*_12_ and 1/*k*_23_, which range from tens of milliseconds to several seconds (Fig. 6). Thus, the activation times estimated herein are in accordance with the previous reports. In addition, the interstate transition rates increase with increasing force (Fig. 6), indicating that force accelerates LFA-1 activation (Fig. 7C)

These results further extend the model for activation of αA domain-containing integrins that we proposed previously [9]. Our molecular dynamics simulations show that applying forces shifted the equilibrium of different conformations of integrin αA domain, which is also supported by the agreement between our mathematical model fits and the experimental data, which indicates that force enhances the transition rates. Without force, the up position of the α_7_-helix in the αA domain is the favored conformation, where the MIDAS ion tends to stay at the outward position, and the ligand binding affinity is low. When force is applied, the equilibrium of the α_7_-helix position is shifted to middle and down; as a result, the MIDAS metal ion tends to stay at the inward position, and the ligand binding affinity is high.

In summary, this study defines the structural basis for mechanical regulation of the kinetics of LFA-1 αA domain conformational changes and relates these simulation results to experimental data of force-induced dissociation of single LFA-1/ICAM-1 bonds by a new mathematical model. Future studies may include simulations to compare αA domains of other integrins and model refinements to add reverse transitions among the three conformational states.

## Methods

### Molecular dynamics simulations

The LFA-1 αA domain was modeled from the crystal structure 1LFA (residues 128–292) [12] except for the distorted α_7_-helix (residues 293–308), which was from another crystal structure 1ZON [11]. The MIDAS Mg^2+^ and all crystallized waters in 1LFA were retained. The modeled structure was soaked in an 80×80×80 Å^3^ water box with periodic boundary conditions, which included 3 Na^+^ and 2 Cl^−^ to neutralize the system. The NAMD package [25] and CHARMM22 all-atom force field [26] were used for energy minimization and molecular dynamics simulations. A 12-Å cutoff was used for van der Waals interactions and Particle Mesh Ewald summation was used to calculate the electrostatic interactions. Energy was minimized in multi-steps with careful treatments of the interactions to avoid any clashes between the α_7_-helix and other portion of the αA domain. The energy-minimized system was then equilibrated for 6 ns with temperature controlled at 310 K by Langevin dynamics with damping coefficient ∼1 ps^−1^ and pressure controlled at 1 atm by Lagevin piston method. At the end of equilibration, the RMSD of the system converged and the α_7_-helix reached a position that aligned well with that observed in the up position of the Mac-1 αA domain structure 1JLM [10]. A 15-ns free dynamics simulation was performed with the equilibrated structure to generate initial conformations for SMD simulations. Two constant-force SMD simulations were performed, starting respectively from 10 and 15 ns of the free dynamics simulations, with the Cα atoms of residues 131–135, 167–172, 177–181 and 232–234 of the β_1_–β_4_ strands harmonically constrained by springs with a spring constant ∼140 pN/Å. A 250-pN force was applied at the C-terminal residue Val308 to pull the α_7_-helix along its axis to the down position suggested by the Mac-1 αA domain structure 1IDO [10]. The backbone hydrogen-bonding atoms in the α_7_-helix were constrained to prevent it from unfolding such that the constraint forces would be added if the distance between the hydrogen-bond pair exceeded 3.5 Å through a spring with a spring constant of ∼700 pN/Å.

With the snapshots obtained from the SMD simulations at 0, 3.7, and 16 ns as respective starting points, we performed additional 50-ns free dynamics simulations for each case, with the Cα atoms of the α_7_-helix residues constrained. At 30 ns, the PSF input file for NAMD was modified such that the point charges of the two carboxyl oxygen atoms of residue D239 were changed from −0.76*e* to −0.26*e*, and the point charge of one Na^+^ atom far away from the protein was changed to 0 to maintain charge neutral of the system.

### Mathematical modeling

We constructed a mathematical model to describe the coupled kinetics of force-induced successive interstate transitions from the three states of LFA-1/ICAM-1 bonds and dissociation from these states (Fig. 4). The three states are denoted as C_1_, C_2_ and C_3_, with interstate transition rates *k*_12_ and *k*_23_ (Fig. 4). Under tensile force, each transition step is assumed to be unidirectional and irreversible, for there was no observable reverse transition of the α_7_-helix position when pulling force was applied (Fig. 2).

The dissociation of the LFA-1/ICAM-1 bond can occur at any of the C_1_, C_2_ and C_3_ states, with intrinsic reverse-rates *k*_r1_, *k*_r2_ and *k*_r3_, respectively. Dissociation from each state is also assumed unidirectional and irreversible. This is reasonable because in the BFP force-clamped experiments [9], once a bond was rupture by tensile force, its component receptor and ligand were pulled apart and no longer able to rebind under the applied force.

Let *p*_1_, *p*_2_ and *p*_3_ denote the respective probabilities of ICAM-1 bound with LFA-1 at C_1_, C_2_ and C_3_ states, respectively. The kinetic equations governing the time evolution of the system can be formulated as:
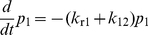
(1)

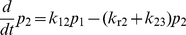
(2)

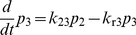
(3)Equations 1–3 can be expressed in a matrix form:
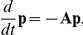
where 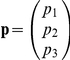, 
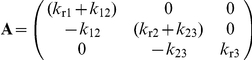


Let *k*_1_, *k*_2_, and *k*_3_ be the eigen-values of **A** with corresponding eigen-vectors **v**_1_, **v**_2_ and **v**_3_, respectively. It can be found that:
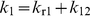
(4)

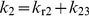
(5)

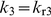
(6)and 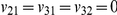, where *v*_ij_ is the *j*th component of the vector **v**_i_. Therefore, the general solution of equations 1–3 can be expressed as:
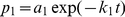
(7)

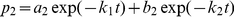
(8)

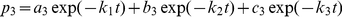
(9)where *a*_1_, *a*_2_, *a*_3_, *b*_2_, *b*_3_, *c*_3_ are nonzero constants. By substituting equations 7–9 into equations 1–3 and compare the corresponding coefficients, we have:
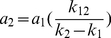
(10)

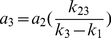
(11)

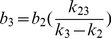
(12)In addition, because both the experimental data [9] and our SMD simulations (Fig. 2) showed that the transition from C_1_ to C_2_ and C_3_ did not happen without force applied, the initial condition can be set as:
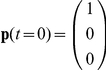
Applying this initial condition to equations 7–9, we have:
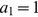
(13)

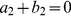
(14)

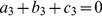
(15)Taking equations 10–15 together, each of *a*_1_, *a*_2_, *a*_3_, *b*_2_, *b*_3_, *c*_3_ can be solved as a function of *k*_12_, *k*_23_, *k*_1_, *k*_2_ and *k*_3_. From this, by letting 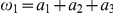, 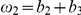, 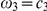, and taking equations 4–6 into account, we got:
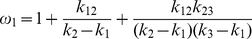
(16)

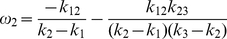
(17)

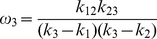
(18)Summing equations 7–9 yields:
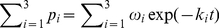
(19)The left hand side of the equation 19 is the total survival probability of the LFA-1/ICAM-1 bond in all states, which corresponds to the measurements from the BFP force-clamped experiments [9]. The format at the right-hand side indicated that *k*_1_, *k*_2_, *k*_3_ should be the apparent off-rates and *ω*_1_, *ω*_2_, *ω*_3_ should be the associated apparent fractions of the three bond states analyzed from the experimental data [9].

With the apparent off-rates *k*_1_, *k*_2_, *k*_3_ and the apparent associated fractions *ω*_1_, *ω*_2_, *ω*_3_ obtained from fitting the experimental data (summarized in Table S1,S2,S3,S4,S5,S6) [9], the intrinsic kinetic parameters *k*_12_, *k*_23_, *k*_r1_, *k*_r2_ and *k*_r3_ can be obtained by solving equations 4–6 and 16–18 and expressed as functions of the known apparent kinetic parameters:
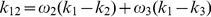
(20)

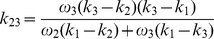
(21)

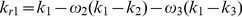
(22)

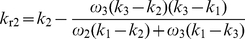
(23)

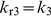
(24)


## Supporting Information

Figure S1 **RMSD time courses of several key elements between the simulated structure and the equilibrated closed (blue) or proposed open (red) conformations of LFA-1 αA domain.** (A) α_7_-helix; (B) MIDAS metal ion Mg^2+^; (C) S139; (D) S141; (E) T206; (F) D239; (G) L289; (H) F292; and (I) L295. The RMSD between the simulated α_7_-helix structure and the equilibrated structure shown in Fig. 2A is redrawn in (A). S139, S141, T206 and D239 are key residues that coordinate the metal ion. L289, F292 and L295 are “ratchet” residues that locate on β6-α7 loop or on α_7_-helix. They have been proposed to be important to the α_7_-helix position.
(TIF)

Table S1 **Model parameters from BFP experiments measured in Mg2+/EGTA condition.**
(DOC)

Table S2 **Model parameters from BFP experiments measured in Ca2+/Mg2+ condition.**
(DOC)

Table S3 **Model parameters from BFP experiments measured in Mn2+ condition.**
(DOC)

Table S4 **Model parameters from BFP experiments measured in Mg2+/EGTA plus XVA143 condition.**
(DOC)

Table S5 **Model parameters from BFP experiments measured in Ca2+/Mg2+ plus XVA143 condition.**
(DOC)

Table S6 **Model parameters from BFP experiments measured in Mn2+ plus XVA143 condition.**
(DOC)

Video S1 **SMD simulation of pulling the α_7_-helix of the LFA-1 αA domain.** The simulated structures were shown in cyan with the α_7_-helix shown in green. The equilibrated α_7_-helix at the up (blue) and down (red) positions are superimposed for comparison. The Mg^2+^ ion is shown as golden spheres. A 250-pN force was applied to the Cα atom of the residue 308 at the C-terminal of the α_7_-helix. At 15 ns, the force was released to allow the system to relax. The Cα atoms of residues 131–135, 167–172, 177–181 and 232–234 of the β_1_–β_4_ strands were constrained to prevent the rigid body motion of the αA domain. The backbone hydrogen-bonding atoms in the α_7_-helix were constrained to prevent it from unfolding.
(MPG)
