# Supplementary material for: Structural Basis and Kinetics of Force-Induced Conformational Changes of an αA Domain-Containing Integrin
Source: PLoS One. 2011 Nov 28;6(11):e27946. doi: 10.1371/journal.pone.0027946 (PMC3225382; doi:10.1371/journal.pone.0027946)
Supplement: Table S1 — Model parameters from BFP experiments measured in Mg2+/EGTA condition. (DOC) [file pone.0027946.s002.doc]

#### Table S1：Model parameters from BFP experiments measured in Mg^2+^/EGTA condition

| ***F* (pN)** | ***k*_1_ (s^-1^)** | ***k*_2_ (s^-1^)** | ***k*_3_ (s^-1^)** | ***ω*_1_** | ***ω*_2_** | ***ω*_3_** |
| --- | --- | --- | --- | --- | --- | --- |
| 0 | 2.78 | - | - | 1 | 0 | 0 |
| 3.06 | 3.24 | 0.548 | - | 0.863 | 0.137 | 0 |
| 5.89 | 4.59 | 0.989 | 0.00541 | 0.714 | 0.198 | 0.0877 |
| 8.89 | 6.16 | 1.64 | 0.0164 | 0.155 | 0.642 | 0.203 |
| 13.0 | 9.44 | 3.21 | 0.0621 | 0.0238 | 0.760 | 0.216 |
| 17.1 | - | 6.78 | 0.343 | 0 | 0.741 | 0.259 |
