# Supplementary material for: Structural Basis and Kinetics of Force-Induced Conformational Changes of an αA Domain-Containing Integrin
Source: PLoS One. 2011 Nov 28;6(11):e27946. doi: 10.1371/journal.pone.0027946 (PMC3225382; doi:10.1371/journal.pone.0027946)
Supplement: Table S2 — Model parameters from BFP experiments measured in Ca2+/Mg2+ condition. (DOC) [file pone.0027946.s003.doc]

#### Table S2：Model parameters from BFP experiments measured in Ca^2+^/Mg^2+^ condition

| ***F* (pN)** | ***k*_1_ (s^-1^)** | ***k*_2_ (s^-1^)** | ***k*_3_ (s^-1^)** | ***ω*_1_** | ***ω*_2_** | ***ω*_3_** |
| --- | --- | --- | --- | --- | --- | --- |
| 0 | 2.96 | - | - | 1 | 0 | 0 |
| 3.72 | 3.34 | 0.460 | - | 0.759 | 0.241 | 0 |
| 6.87 | 5.77 | 0.663 | 0.00930 | 0.596 | 0.284 | 0.120 |
| 10.4 | 8.74 | 0.931 | 0.0255 | 0.277 | 0.537 | 0.186 |
| 15.1 | 14.6 | 3.70 | 0.0568 | 0.246 | 0.613 | 0.142 |
| 18.8 | 20.5 | 6.63 | 0.424 | 0.185 | 0.702 | 0.112 |
