# Supplementary material for: Structural Basis and Kinetics of Force-Induced Conformational Changes of an αA Domain-Containing Integrin
Source: PLoS One. 2011 Nov 28;6(11):e27946. doi: 10.1371/journal.pone.0027946 (PMC3225382; doi:10.1371/journal.pone.0027946)
Supplement: Table S3 — Model parameters from BFP experiments measured in Mn2+ condition. (DOC) [file pone.0027946.s004.doc]

#### Table S3：Model parameters from BFP experiments measured in Mn^2+^ condition

| ***F* (pN)** | ***k*_1_ (s^-1^)** | ***k*_2_ (s^-1^)** | ***k*_3_ (s^-1^)** | ***ω*_1_** | ***ω*_2_** | ***ω*_3_** |
| --- | --- | --- | --- | --- | --- | --- |
| 0 | 2.66116 | - | - | 1 | 0 | 0 |
| 2.5 | 3.27025 | 0.38655 | 0.002037 | 0.813916 | 0.179137 | 0.006947 |
| 6 | 5.91536 | 0.555514 | 0.0052033 | 0.637665 | 0.342294 | 0.020041 |
| 10 | 7.32381 | 0.949709 | 0.0138781 | 0.357543 | 0.431565 | 0.210892 |
| 14 | 10.41546 | 1.78496 | 0.0374057 | 0.141697 | 0.52644 | 0.331863 |
| 19 | 18.56965 | 3.24104 | 0.163543 | 0.092277 | 0.53384 | 0.373883 |
