# Supplementary material for: Structural Basis and Kinetics of Force-Induced Conformational Changes of an αA Domain-Containing Integrin
Source: PLoS One. 2011 Nov 28;6(11):e27946. doi: 10.1371/journal.pone.0027946 (PMC3225382; doi:10.1371/journal.pone.0027946)
Supplement: Table S4 — Model parameters from BFP experiments measured in Mg2+/EGTA plus XVA143 condition. (DOC) [file pone.0027946.s005.doc]

#### Table S4：Model parameters from BFP experiments measured in Mg^2+^/EGTA plus XVA143 condition

| ***F* (pN)** | ***k*_1_ (s^-1^)** | ***k*_2_ (s^-1^)** | ***k*_3_ (s^-1^)** | ***ω*_1_** | ***ω*_2_** | ***ω*_3_** |
| --- | --- | --- | --- | --- | --- | --- |
| 0 | 2.60552371 | 0.361259 | 0.001075005 | 1 | 0 | 0 |
| 2.94 | 3.4653502 | 0.588534 | 0.002726946 | 0.942063 | 0.057937 | 0 |
| 6.47 | 4.88038298 | 1.057448 | 0.008338169 | 0.921359 | 0.078641 | 0 |
| 9.46 | 6.52247363 | 1.737068 | 0.021488792 | 0.901482 | 0.098518 | 0 |
| 13.1 | 9.28438257 | 3.178594 | 0.068034916 | 0.736075 | 0.263925 | 0 |
