# Supplementary material for: Structural Basis and Kinetics of Force-Induced Conformational Changes of an αA Domain-Containing Integrin
Source: PLoS One. 2011 Nov 28;6(11):e27946. doi: 10.1371/journal.pone.0027946 (PMC3225382; doi:10.1371/journal.pone.0027946)
Supplement: Table S5 — Model parameters from BFP experiments measured in Ca2+/Mg2+ plus XVA143 condition. (DOC) [file pone.0027946.s006.doc]

#### Table S5：Model parameters from BFP experiments measured in Ca^2+^/Mg^2+^ plus XVA143 condition

| ***F* (pN)** | ***k*_1_ (s^-1^)** | ***k*_2_ (s^-1^)** | ***k*_3_ (s^-1^)** | ***ω*_1_** | ***ω*_2_** | ***ω*_3_** |
| --- | --- | --- | --- | --- | --- | --- |
| 0 | 2.65816055 | 0.20305 | 0.000515225 | 1 | 0 | 0 |
| 4.61 | 4.41379279 | 0.463423 | 0.00241379 | 0.925275 | 0.074725 | 0 |
| 8.08 | 6.46520268 | 0.862445 | 0.007718717 | 0.855445 | 0.144555 | 0 |
| 12.56 | 10.5828457 | 1.923095 | 0.034620577 | 0.694664 | 0.305336 | 0 |
| 17.21 | 17.6499749 | 4.420647 | 0.16438286 | 0.474277 | 0.525723 | 0 |
