# Supplementary material for: Structural Basis and Kinetics of Force-Induced Conformational Changes of an αA Domain-Containing Integrin
Source: PLoS One. 2011 Nov 28;6(11):e27946. doi: 10.1371/journal.pone.0027946 (PMC3225382; doi:10.1371/journal.pone.0027946)
Supplement: Table S6 — Model parameters from BFP experiments measured in Mn2+ plus XVA143 condition. (DOC) [file pone.0027946.s007.doc]

#### Table S6：Model parameters from BFP experiments measured in Mn^2+^ plus XVA143 condition

| ***F* (pN)** | ***k*_1_ (s^-1^)** | ***k*_2_ (s^-1^)** | ***k*_3_ (s^-1^)** | ***ω*_1_** | ***ω*_2_** | ***ω*_3_** |
| --- | --- | --- | --- | --- | --- | --- |
| 0 | 2.646384774 | 0.276686 | 0.001059 | 1 | 0 | 0 |
| 7 | 5.593663685 | 0.666999 | 0.006641 | 0.912492 | 0.087508 | 0 |
| 12 | 9.547071634 | 1.250494 | 0.024647 | 0.828085 | 0.171915 | 0 |
